# Neoadjuvant triplet immune checkpoint blockade in newly diagnosed glioblastoma

**DOI:** 10.1038/s41591-025-03512-1

**Published:** 2025-02-27

**Authors:** Georgina V. Long, Elena Shklovskaya, Laveniya Satgunaseelan, Yizhe Mao, Inês Pires da Silva, Kristen A. Perry, Russell J. Diefenbach, Tuba N. Gide, Brindha Shivalingam, Michael E. Buckland, Maria Gonzalez, Nicole Caixeiro, Ismael A. Vergara, Xinyu Bai, Robert V. Rawson, Edward Hsiao, Umaimainthan Palendira, Tri Giang Phan, Alexander M. Menzies, Matteo S. Carlino, Camelia Quek, Sean M. Grimmond, Joseph H. A. Vissers, Dannel Yeo, John E. J. Rasko, Mustafa Khasraw, Bart Neyns, David A. Reardon, David M. Ashley, Helen Wheeler, Michael Back, Richard A. Scolyer, James Drummond, James S. Wilmott, Helen Rizos

**Affiliations:** 1 Melanoma Institute Australia, University of Sydney, Sydney, New South Wales Australia; 2 Faculty of Medicine and Health, University of Sydney, Sydney, New South Wales Australia; 3 Charles Perkins Centre, University of Sydney, Camperdown, New South Wales Australia; 4 Mater Hospital, North Sydney, New South Wales Australia; 5 Royal North Shore Hospital, St Leonards, New South Wales Australia; 6 Macquarie University, Macquarie Park, New South Wales Australia; 7 Royal Prince Alfred Hospital, Camperdown, New South Wales Australia; 8 Chris O’Brien Lifehouse, Camperdown, New South Wales Australia; 9 NSW Health Pathology, Sydney, New South Wales Australia; 10 Garvan Institute of Medical Research, Darlinghurst, New South Wales Australia; 11 Blacktown Hospital, Blacktown, New South Wales Australia; 12 Westmead Hospital, Westmead, New South Wales Australia; 13 Collaborative Centre for Genomic Cancer Medicine, University of Melbourne, Melbourne, Victoria Australia; 14 Department of Clinical Pathology, University of Melbourne, Melbourne, Victoria Australia; 15 Centenary Institute, Camperdown, New South Wales Australia; 16 Duke University, Durham, NC USA; 17 Universitair Ziekenhuis Brussel, Brussels, Belgium; 18 Center for Neuro-Oncology, Dana-Farber Cancer Institute, Boston, MA USA; 19 Sydney Medical School, University of Sydney, Sydney, New South Wales Australia; 20 North Shore Radiology & Nuclear Medicine, St Leonards, New South Wales Australia; 21 Brain Imaging Laboratory, The Brain Cancer Group, St Leonards, New South Wales Australia

**Keywords:** CNS cancer, Tumour immunology

## Abstract

Glioblastoma (GBM) is an aggressive primary adult brain tumor that rapidly recurs after standard-of-care treatments, including surgery, chemotherapy and radiotherapy. While immune checkpoint inhibitor therapies have transformed outcomes in many tumor types, particularly when used neoadjuvantly or as a first-line treatment, including in melanoma brain metastases, they have shown limited efficacy in patients with resected or recurrent GBM. The lack of efficacy has been attributed to the scarcity of tumor-infiltrating lymphocytes (TILs), an immunosuppressive tumor microenvironment and low tumor mutation burden typical of GBM tumors, plus exclusion of large molecules from the brain parenchyma. We hypothesized that upfront neoadjuvant combination immunotherapy, administered with disease in situ, could induce a stronger immune response than treatment given after resection or after recurrence. Here, we present a case of newly diagnosed IDH*-*wild-type, *MGMT* promoter unmethylated GBM, treated with a single dose of neoadjuvant triplet immunotherapy (anti-programmed cell death protein 1 plus anti-cytotoxic T-lymphocyte protein 4 plus anti-lymphocyte-activation gene 3) followed by maximal safe resection 12 days later. The anti-programmed cell death protein 1 drug was bound to TILs in the resected GBM and there was marked TIL infiltration and activation compared with the baseline biopsy. After 17 months, there is no definitive sign of recurrence. If used first line, before safe maximal resection, checkpoint inhibitors are capable of immune activation in GBM and may induce a response. A clinical trial of first-line neoadjuvant combination checkpoint inhibitor therapy in newly diagnosed GBM is planned (GIANT; trial registration no. NCT06816927).

## Abstract (web summary)

A patient with newly diagnosed glioblastoma was safely treated with neoadjuvant nivolumab, relatlimab and ipilimumab before maximal resection, with comprehensive immune profiling showing the induction of overall immune activation early during treatment. The patient had no definitive evidence of recurrence at 17 months after treatment.

## Main

Glioblastoma (GBM) is the most aggressive form of adult-type diffuse glioma and is characterized by the absence of *IDH1* and *IDH2* mutations^1^. Standard therapy, of maximal safe resection followed by radiotherapy and temozolomide chemotherapy (the Stupp protocol^2^), provides a 2.5-month median overall survival (OS) benefit over surgery with radiotherapy alone in unselected IDH-agnostic patients with GBM^2^. Patients with *MGMT* promoter-unmethylated GBM have the worst outcomes, with a median OS of 14.1 months despite standard-of-care chemoradiotherapy^3^. Most patients will die within the first 2 years of diagnosis^3^.

Immune checkpoint inhibitors (ICIs) have revolutionized the management of many cancer types, including melanoma, where the 10-year melanoma-specific survival rate has increased from less than 5% to 52% of patients in stage IV treated with combination ICIs^4^. In randomized GBM trials, adjuvant anti-programmed cell death protein 1 (PD-1) administered in combination with radiotherapy (plus temozolomide in methylated GBM) after maximal safe resection and followed by anti-PD-1 monotherapy to 12 months, showed no benefit over the standard Stupp protocol^5–7^. The lack of ICI activity in GBM is thought to reflect a low tumor mutational burden (TMB) and an immunosuppressive tumor microenvironment (TME) characterized by abundant microglia and macrophages, a scarcity of tumor-infiltrating lymphocytes (TILs) and immature natural killer cells^8,9^.

Neoadjuvant ICI outperforms adjuvant delivery in many cancer types but has only been tested using single-agent anti-PD-1 in small numbers of heavily pretreated (corticosteroids and chemoradiotherapy) patients with recurrent GBM^10–13^ (Extended Data Table 1). In these patients, neoadjuvant PD-1 blockade promoted the activation of TILs^10–12^ and extended OS (13.7 months with neoadjuvant ICI versus 7.5 months with adjuvant)^11^. Notably, two of three newly diagnosed patients with GBM who received neoadjuvant anti-PD-1 before maximal safe resection remained disease free for more than 33 and 28 months, respectively^12^. Response to neoadjuvant immunotherapy may be supported by the presence of tumor-associated effector CD8^+^ T cells identified in the cranial bone marrow of treatment-naive, newly diagnosed patients with GBM^14^.

Here, we describe a case study of a newly diagnosed patient with GBM (IDH-wild-type, unmethylated *MGMT* promoter) treated with triplet neoadjuvant ICI upfront (nivolumab, anti-PD-1; ipilimumab, anti-cytotoxic T-lymphocyte protein 4 (CTLA-4); and relatlimab, anti-lymphocyte-activation gene 3 (LAG3)), before maximal safe resection. Triple ICI therapy was selected based on an unprecedented survival in the phase 1 trial in advanced melanoma^15^, as well as the significantly superior activity of the combination of multiple ICIs versus ICI monotherapy in melanoma with a poor TME^16,17^ (low CD8^+^ T cells, low TMB and low interferon-γ (IFNγ)), metastases to the brain^18^ or primary resistance to anti-PD-1 therapy^19^—all features found in GBM. Our findings confirm the urgent need to investigate this strategy in clinical trials.

## Patient and GBM characteristics

**Fig. 1:**
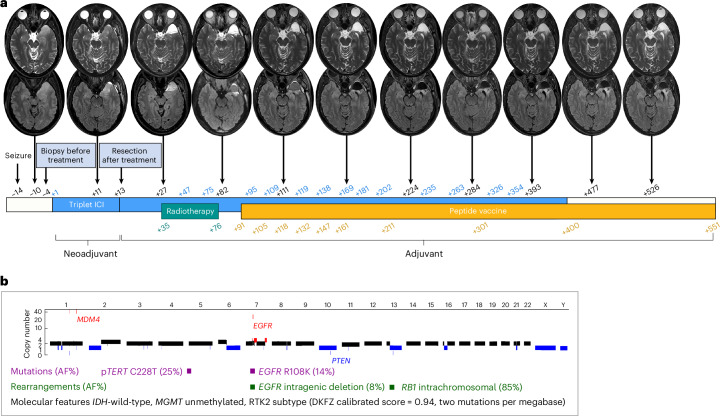
Clinical and molecular details of the patient with GBM and treatment regimen. **a**, Serial magnetic resonance imaging scans with gadolinium including T2-weighted (top) and fluid-attenuated inversion recovery sequences (bottom) shown during the treatment schedule. The treatment time points, relative to neoadjuvant ICI therapy initiation (day +1) are shown, including ICI cycles (blue), radiotherapy (green) and peptide vaccine doses (yellow). The peptide vaccine program was recently completed, and ongoing analyses will be reported separately. The most recent scan (day +526) demonstrated grossly stable postsurgical changes with persistent white matter T2 fluid-attenuated inversion recovery hyperintensity in the left temporal periventricular white matter posterior to the temporal pole surgical bed and the left temporal operculum. There was a stable small focus of enhancement lateral to the left temporal horn. These changes were all within the high-dose radiotherapy field. No focal hyperperfusion or diffusion restriction to suggest recurrent disease was observed. **b**, Key genomic events identified in pretreatment GBM. Segment-level copy number profile across chromosomes includes amplifications in red (copy number > 2.5) and deletions in blue (copy number < 1.5). Copy number-neutral regions are shown in black. For copy number, the *y* axis is shown in pseudo-logarithmic scale. Somatic mutations (magenta) and rearrangements (dark green) of potential clinical significance or in known cancer genes are included in the genomic region where each gene resides together with their allele fraction (AF). All events were identified based on the WGS of the pretreatment (day –4) tumor specimen, except for the *EGFR* (R108K) mutation identified based on the WGS of the resection (posttreatment; day +13) specimen.

A previously well 56-year-old male presented with new-onset generalized seizure and no prior symptoms (pre-ICI treatment, day −14). After initial management (Supplementary Results 1), the patient was diagnosed with a left temporal lobe GBM (IDH-wild-type, central nervous system WHO grade 4, RTK2 subtype (DKFZ calibrated score = 0.94)) after an open craniotomy biopsy (pre-ICI treatment, day −4; Fig. 1a). The tumor was a pleomorphic, mitotically active, diffusely infiltrative astrocytic glioma with negative IDH1 (R132H) immunohistochemistry (IHC) and retained nuclear ATRX stain. Mitoses were present at up to 8–10 per ten high-power fields (field diameter = 0.58 mm). There was no evidence of microvascular proliferation or necrosis (Supplementary Results 2). Pyrosequencing revealed a *TERT* promoter mutation (C228T) and confirmed no *IDH1* codon 132 or *IDH2* codon 172 mutation. The tumor was unmethylated at the *MGMT* promoter region. Moderate-to-strong p53 nuclear staining was observed in tumor cells. Microsatellite status was stable. Whole-genome sequencing (WGS) (mean coverage = 85X) revealed focal amplification of the *EGFR* and *MDM4* genes, homozygous *PTEN* deletion and inactivating rearrangement of *RB1* with a low TMB (two mutations per megabase; Fig. 1b and Extended Data Table 2).

The patient’s Eastern Cooperative Oncology Group Performance Status fluctuated between 0 and 1 because of postictal symptoms, surgery and lumbar punctures in the initial 4 weeks after presentation. In the 2 weeks before the initiation of treatment, there was no evidence of tumor growth or peritumoral edema on imaging. Corticosteroids were not commenced at any time point.

## Neoadjuvant combined (triplet) ICI and outcomes

**Extended Data Fig. 1:**
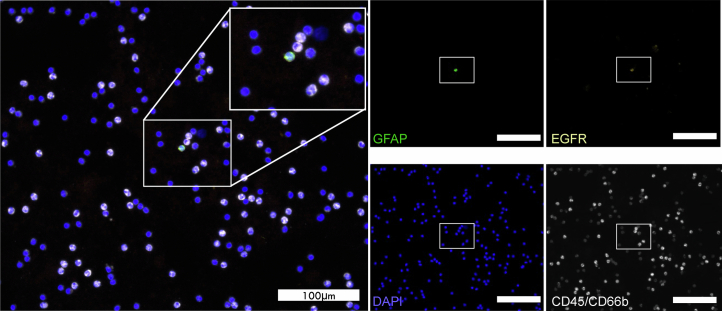
Identification of circulating tumor cells. Representative region of whole-slide immunofluorescence showing circulating tumor cell (CTC, boxed) identified in a GBM patient. Cells were stained for GFAP (green), EGFR (yellow), CD45/CD66b (white) and nuclear DAPI (blue). Magnification, 40X. Sample collected pretreatment (day –9). 7.5 mL peripheral blood was processed on the AccuCyte-CyteFinder platform (RareCyte). Nucleated blood cells were spread on 8 slides. Slides were stained using DAKO autostainer and the whole slide was imaged and analyzed by CyteMapper software for DAPI ^+^ GFAP ^+^ EGFR^ + /−^ CD45/CD66b^−^ cells with user verification. Scale bars = 100 μm. Source data

After treatment with intravenous neoadjuvant triplet ICI on day +1 (cycle 1: nivolumab 480 mg plus relatlimab 160 mg plus ipilimumab 80 mg), the patient underwent safe maximal resection (day +13; Supplementary Results 3), a 6-week course of adjuvant radiotherapy from day +35 (60 Gy in 30 fractions), adjuvant ICI from day +47 and adjuvant personalized peptide vaccination from day +91 (Fig. 1 and Extended Data Table 3). Adjuvant ICIs were administered as monotherapy or in combination, depending on toxicity and with avoidance of corticosteroids (grade 1 (and after cycle 12, grade 3) hepatitis, grade 1 conjunctivitis and grade 1 dermatitis attributed to ipilimumab). Circulating tumor cells (CTCs) (4′,6-diamidino-2-phenylindole (DAPI) + glial fibrillary acidic protein (GFAP) + epidermal growth factor receptor (EGFR) + CD45^−^CD66b^−^) zero-converted after therapy (16 CTCs in 7.5 ml blood detected on day −9 versus zero CTCs on day +190; Extended Data Fig. 1). At the last radiographic assessment (day +526, 17 months), there was no definitive evidence of recurrence (Fig. 1a).

## Neoadjuvant ICI reshapes the GBM immune landscape

**Fig. 2:**
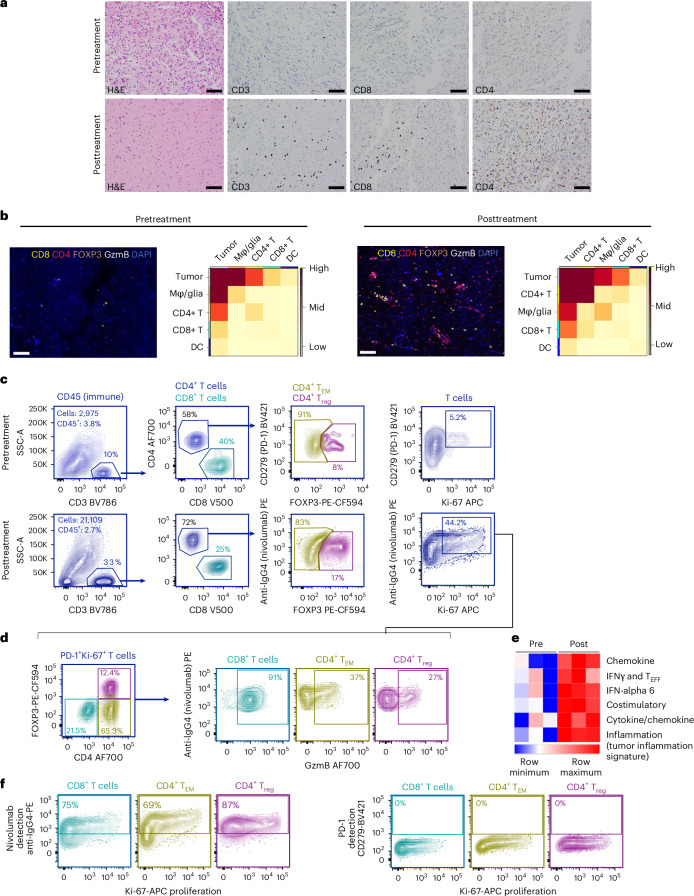
Evaluation of immune cells in the GBM tumor before (day −4) and after (day +13) neoadjuvant ICI treatment. **a**, Representative region of whole-slide hematoxylin and eosin (H&E) and IHC images of paired pretreatment and posttreatment GBM specimens for CD3^+^, CD8^+^ and CD4^+^ T cells. Representative images were taken from areas of increased tumor cellularity. **b**, Representative region of whole-slide high-plex immunofluorescence images and cellular neighborhood enrichment analysis showing the co-occurrence of specific immune cells (CD8^+^ T and CD4^+^ T cells, dendritic cells (DCs), macrophages and glia) with tumor cells in the paired pretreatment and posttreatment GBM specimens. One slide was prepared and representative images were taken from areas of increased tumor cellularity. **c**, The tumor-infiltrating CD45^+^ (immune) fraction was analyzed for T cell content (CD3^+^), T cell subsets (CD8^+^, CD4^+^FOXP3^−^ T_EM_ and CD4^+^FOXP3^+^ T_reg_ cells) and activation markers (PD-1^+^Ki-67^+^). PD-1 expression on T cells was detected with anti-PD-1-Brilliant Violet 421 (BV421) in the pretreatment tumor or anti-IgG4-PE (detects nivolumab bound to PD-1) in the posttreatment tumor. **d**, The fraction of activated and proliferating T cell subsets, along with their GzmB expression, were evaluated in the posttreatment GBM. There was insufficient material available for analysis of the T cell subsets in the pretreatment biopsy specimen. **e**, Heatmap showing the immune activation transcriptome gene set^36^ scores (derived from the singscore method^37^) in the pretreatment and posttreatment tumor specimens (RNA was analyzed in triplet for each tumor specimen using the NanoString PanCancer IO360 Panel). **f**, The posttreatment tumor was analyzed for drug/nivolumab occupancy of PD-1 sites (nivolumab detection, staining with anti-IgG4-PE) and residual unoccupied PD-1 sites (PD-1 detection, staining with anti-PD-1 (CD279)-BV421). Scale bars = 100 µm. Source data

**Extended Data Fig. 2:**
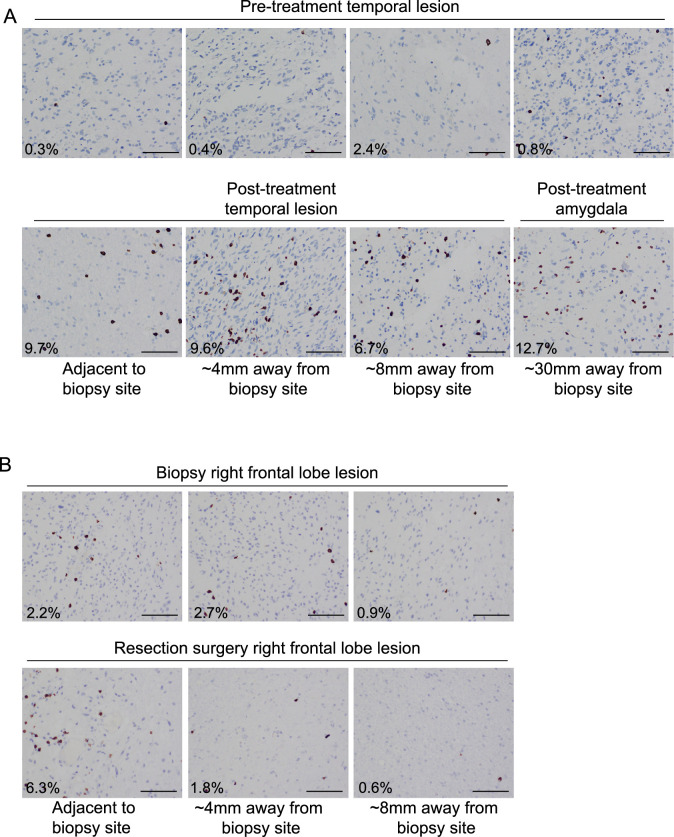
Evaluation of CD3+ immune cells in longitudinal GBM biopsy and surgical specimens in A) the ICI-treated left temporal GBM and B) an untreated right frontal GBM. **a**) Representative region of whole-slide immunohistochemistry images of pretreatment (day –4) and posttreatment (day +13) GBM specimens for CD3^+^ T cells across left temporal and amygdala sections. Percentage CD3^+^ T cell content is shown and was quantitated from over 1,300 cells in each tissue specimen using QuPath. Representative regions were taken from areas of increased tumor cellularity. **b**) Representative region of whole-slide immunohistochemistry images showing CD3^+^ T cell infiltration in biopsy and resection specimens from a 66-year-old male with right frontal lobe GBM. The patient underwent an initial diagnostic biopsy, followed by maximal tumor resection 14 days later. Percentage CD3^+^ T cell content is shown and was quantitated from over 1,200 cells in each tissue specimen using QuPath. Representative regions were taken from areas of increased tumor cellularity. Scale bars = 100 μm. Source data

Chromogenic IHC and high-plex immunofluorescence of pretreatment (day −4) and posttreatment (day +13) formalin-fixed paraffin-embedded tumor specimens confirmed increases in the number of CD3^+^ T cells (Fig. 2a,b), including CD3^+^CD4^+^ T cells (greater than tenfold increase in the posttreatment specimen relative to pretreatment (from 0.3% to 5%)) and CD3^+^CD8^+^ cytotoxic T cells (eightfold increase posttreatment relative to pretreatment (from 0.4% to 3%); Fig. 2b). To account for potential localized inflammation induced by the initial biopsy, we confirmed a global increase in infiltration of CD3^+^ T cells via chromogenic IHC across all posttreatment tumor sections analyzed from the left temporal and amygdala regions (up to 30 mm from the biopsy site), compared with the pretreatment biopsy tissue (Extended Data Fig. 2). This contrasted with our findings in a resection sample from a separate patient with an untreated right frontal GBM, showing only a very localized T cell infiltrate due to the diagnostic biopsy performed 14 days before the resection (nearly zero T cells 8 mm from the biopsy site; Extended Data Fig. 2). High-dimensional-spatial phenotyping of formalin-fixed paraffin-embedded GBM specimens revealed increased colocalization of CD4^+^ and CD8^+^ T cells with tumor cells, and a notable decrease in the colocalization of macrophages and microglial cells with tumor cells in response to neoadjuvant ICI treatment (Fig. 2b).

**Extended Data Fig. 3:**
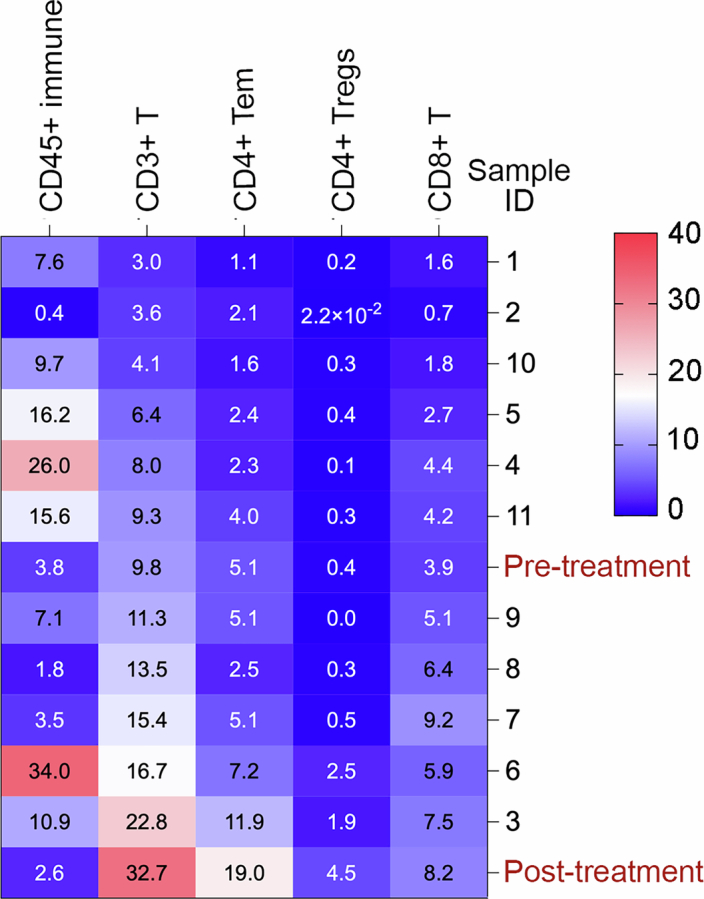
Frequency of lymphocyte subsets in primary GBM. Heat map showing frequency of lymphocyte subsets in primary GBM tumor dissociates (n = 13), including the pretreatment and posttreatment GBM tumor dissociates (highlighted in red). Samples are ranked according to CD3^+^ T cell content. T cell subsets (CD3^+^ T cells, CD4^+^FOXP3^+^ regulatory T cells (Tregs), CD4^+^FOXP3^-^ effector memory T cells (Tem) and CD8^+^ T cells) are shown as percentage of total CD45^+^ cells. CD45^+^ lymphocytes are shown as percentage of viable cells. Source data

Multiparameter flow cytometry analysis was performed on the freshly dissociated pretreatment and posttreatment GBM specimens, and on an unrelated series of 11 primary GBM tumors resected from patients undergoing their first surgery. The CD45^+^ immune cell content in the 12 pretreatment GBMs ranged from 0.4% to 34.0% of the total viable cell population (median = 8.7%; Extended Data Fig. 3). The frequency of tumor-infiltrating CD3^+^ (range = 3–23%; median = 9.5%), CD4^+^FOXP3^−^ effector memory (T_EM_) (range = 1–12%; median = 3.2%), CD4^+^FOXP3^+^ regulatory T (T_reg_) cells (range = 0–2.5%; median = 0.3%) and CD8^+^ T cells (range = 1–9%; median = 4.3%) were also variable in the pretreatment GBM specimens (Extended Data Fig. 3). After ICI treatment, the posttreatment GBM specimen showed a marked increase in the relative proportion of T cells, with CD3^+^ T cells rising from 9.8% to 32.7%, CD4^+^ T_EM_ cells from 5.1% to 19.0%, CD4^+^ T_reg_ cells from 0.4% to 4.5% and CD8^+^ T cells from 3.9% to 8.2% (Extended Data Fig. 3). When compared with 31 pretreatment primary GBM tumors (12 from this study and 19 from a published series^20^), the ICI-treated posttreatment GBM specimen had the highest proportion of infiltrating CD3^+^ and CD4^+^ T cells and among the highest frequency of infiltrating CD8^+^ T cells.

**Fig. 3:**
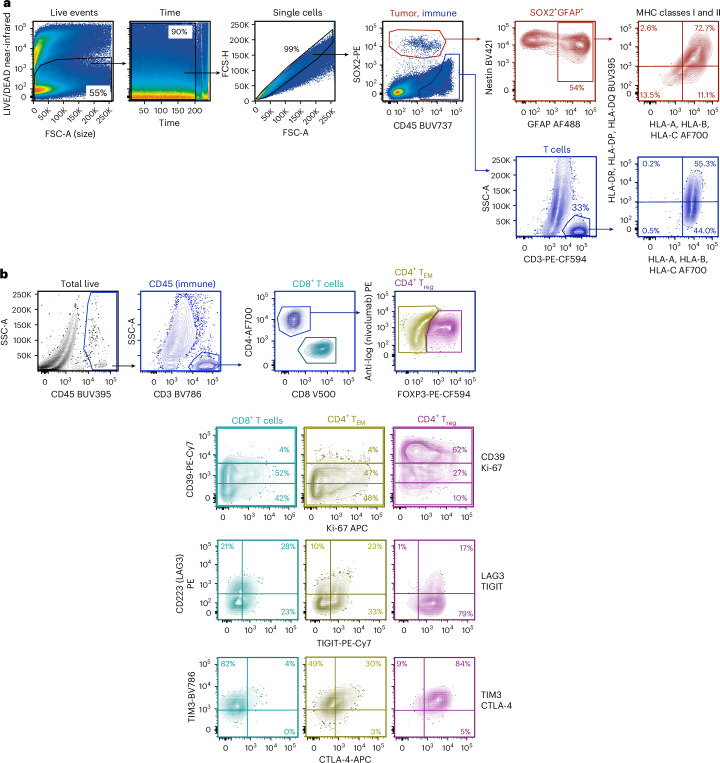
Flow cytometry tumor gating strategy and immune checkpoint analysis. **a**, General gating strategy for tumor dissociates. Left to right, a viability gate to exclude dead cells, a time gate to exclude electronic noise, a singlet gate to exclude doublets and tumor and immune fraction gates to define the cells of interest. Tumor cells (SOX2^+^) were further gated for the GFAP^+^ fraction and analyzed for MHC class I (HLA-A, HLA-B, HLA-C) and MHC class II (HLA-DR, HLA-DP, HLA-DQ) expression. The numbers indicate the percentage of cells in the respective gates. T cell analysis is shown for comparison. **b**, The tumor-infiltrating CD45^+^ (immune) fraction was analyzed for T cell content (CD3^+^), T cell subsets (CD8^+^ T, CD4^+^FOXP3^−^ T_EM_ and CD4^+^FOXP3^+^ T_reg_ cells), and immune checkpoints (CD39, LAG3, TIGIT, TIM3 and CTLA-4). The numbers indicate the percentage of cells in the respective gates. BUV, brilliant ultra-violet; FSC, forward scatter; FSC-A, forward scatter area; FSC-H, forward scatter height; SSC, side scatter; SSC-A, side scatter area. Source data

**Fig. 4:**
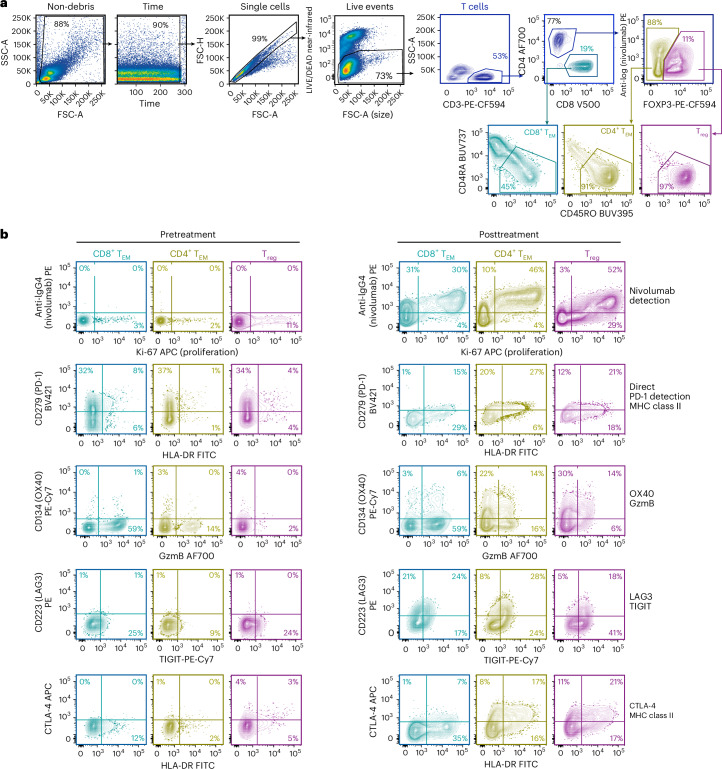
Evaluation of circulating T cells before and after neoadjuvant ICI treatment. **a**, General gating strategy for PBMC samples. Left to right, a debris exclusion gate, a time gate to exclude electronic noise, a singlet gate to exclude doublets and a viability gate to exclude dead cells. T cells (CD3^+^ SSC-A-low) were gated for CD45RA^−^CD45RO^+^ effector and memory subsets: CD8^+^ T_EM_ (CD3^+^CD8^+^CD45RA^−^CD45RO^+^), CD4^+^ T_EM_ (CD3^+^CD4^+^FOXP3^−^CD45RA^−^CD45RO^+^) and T_reg_ (CD3^+^CD4^+^FOXP3^+^CD45RA^–^CD45RO^+^) cells. **b**, CD8^+^ T_EM_, CD4^+^ T_EM_ and T_reg_ subsets in the pretreatment (day −4, left) and posttreatment (day +12, right) samples were analyzed for (top to bottom): T cell-bound nivolumab (detected with anti-IgG4-PE), residual unoccupied PD-1 (direct PD-1 detection with anti-PD-1 (CD279) BV421), activation markers (MHC class II, OX40) and GzmB. Immune checkpoint (LAG3, CTLA-4, TIGIT) expression analysis was performed on pretreatment (day −9) samples. The numbers indicate the percentage of cells in the respective gates. Source data

Further analyses of the paired pretreatment and posttreatment GBM revealed that the activated (PD-1^+^Ki-67^+^) tumor-infiltrating immune fraction increased 8.5-fold (from 5.2% to 44.2%) after neoadjuvant ICI therapy and was dominated by CD4^+^ T_EM_ cells (65.3%), followed by CD8^+^ (21.5%) and T_reg_ cells (12.4%; Fig. 2c,d). Activated granzyme B (GzmB)-expressing CD8^+^, CD4^+^ T_EM_ and CD4^+^ T_reg_ cells were also abundant (91%, 37% and 27%, respectively) in the posttreatment tumor (Fig. 2d). Transcriptome signatures indicative of immune cell activation, including IFNγ, chemokine, costimulatory and tumor inflammation gene sets were all elevated in the GBM tumor after ICI treatment (Fig. 2e). These changes in immune cell activity corresponded with complete occupancy of PD-1 by nivolumab on tumor-infiltrating CD8^+^ T, CD4^+^ T_EM_ and CD4^+^ T_reg_ cells in response to treatment (Fig. 2f). The expression of tumor major histocompatibility complex (MHC) classes I and II was examined after treatment; 83.8%, 75.3% and 72.7% of GFAP^+^/SOX2^+^ GBM cells expressed MHC class I, MHC class II or both MHC classes I and II, respectively (Fig. 3a). The immune checkpoints—LAG3, TIM3, CTLA-4, TIGIT and CD39—were all highly expressed in tumor-infiltrating T cells after treatment (Fig. 3b). Although there was insufficient pretreatment biopsy material to confirm checkpoint induction in response to ICI therapy in infiltrating immune cells, checkpoint expression was potently induced from before to after neoadjuvant therapy in peripheral blood cell subsets (Fig. 4a,b).

**Fig. 5:**
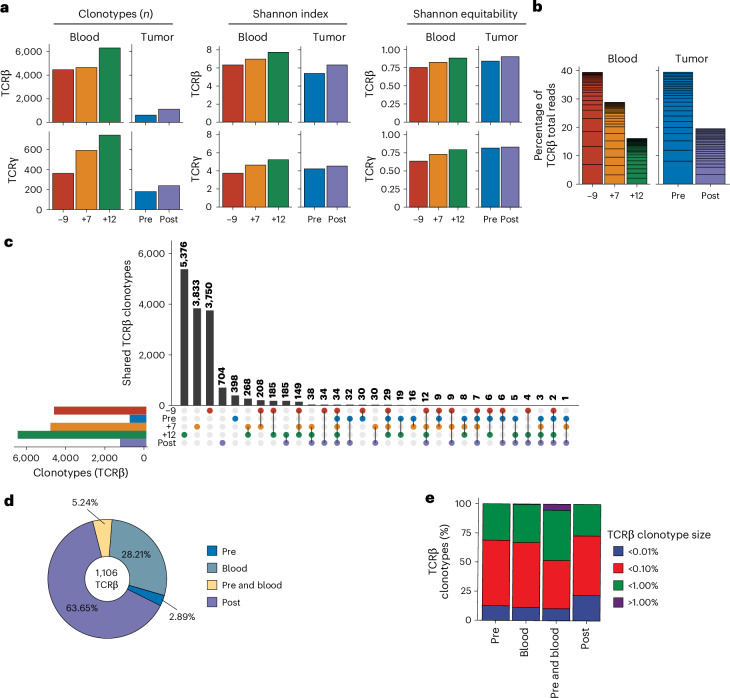
Neoadjuvant ICI-induced TCR repertoire and immune cell changes. **a**, TCRβ and TCRγ clone numbers, Shannon diversity index and Shannon equitability index scores (measurement of the similarity of clone sizes) increased after neoadjuvant ICIs in the tumor (pretreatment (day −4) and posttreatment (day +13)) and blood specimens (pretreatment (day −9) and posttreatment (days +7 and +12)). **b**, The TCR clone size (clonotype total reads expressed as a percentage of total productive reads) of the 25 largest TCR clonotypes in each sample. The largest clones are at the bottom of each column. **c**, Upset plot of the shared TCRβ clonotypes across tumor (before and after) and blood specimens (day −9, day +7, day +12). The vertical bars indicate the number of clonotypes; the sample distribution patterns are indicated below the chart and are represented by filled points linked by the lines. A filled circle indicates that clones were detected in the corresponding samples; a gray circle indicates that TCR clones were not detected. The first five vertical bars show clonotypes unique to each of the specimens. The horizontal bars (lower left) show the number of TCRβ clones in each sample. **d**, Tracking the origin of clonotypes detected in the posttreatment tumor specimen. TCRβ clonotypes were classified based on whether they were unique to the posttreatment sample (63.65% of clonotypes) or shared with any of the PBMC samples (blood), the pretreatment tumor sample or both the pretreatment tumor and blood. The number in the center indicates the number of productive TCRβ clonotypes in the posttreatment tumor sample. **e**, Details of the size (clonotype total reads/sample total reads expressed as a percentage) of TCRβ clonotypes identified in the posttreatment tumor according to their origin (that is, identified in PBMC sample (blood), the pretreatment tumor sample or both the pretreatment tumor and blood samples). Most expanded clones (accounting for more than 1% of the sample total reads) were shared with the pretreatment tumor and blood samples. Source data

The number of productive T cell receptor (TCR)β and TCRγ clonotypes and the Shannon diversity index of TCRβ and TCRγ increased in the posttreatment tumor sample compared with the pretreatment specimen (Fig. 5a). Increased TCR diversity was associated with increased Shannon equitability (that is, similarity of clone frequency; Fig. 5a), confirming a more diverse TCR repertoire with reduced TCR clone dominance after neoadjuvant ICI therapy (Fig. 5b). TCR clone sharing analysis confirmed that 63.65% of TCRβ clones were uniquely identified in the tumor after treatment, whereas 28.21% of TCRβ clones infiltrating the tumor after ICI therapy were also detected in the circulation (Fig. 5c,d). Importantly, TCRβ clones shared between the pretreatment, posttreatment and peripheral blood mononuclear cell (PBMC) samples were the most expanded clones (that is, TCR clones with more than 1% of the sample total TCR reads; Fig. 5e). These data suggest early clonal expansion of preexisting clonotypes.

## Dynamic changes in peripheral immune markers

Analysis of circulating TCRβ and TCRγ in PBMC samples collected before treatment (day −9) and after treatment (days +7 and +12) confirmed an increasing number, diversity and equitability of TCR clones after neoadjuvant ICI therapy (Fig. 5a).

Peripheral blood cell subsets collected before treatment (day −9) and after treatment (day +12) with neoadjuvant ICI therapy were analyzed using flow cytometry. The posttreatment sample contained substantially higher percentages of proliferating effector and memory (CD45+RA^−^RO^+^) CD8^+^, CD4^+^ T_EM_ and CD4^+^ T_reg_ (Ki-67^+^ fraction 34%, 50% and 81%, respectively; up from 3%, 2% and 11% in the pretreatment blood samples; Fig. 4b) cell subsets. The activation markers OX40 and MHC class II, and the cytotoxic marker GzmB, were each induced in circulating CD8^+^, CD4^+^ and T_reg_ subsets after neoadjuvant ICI therapy; PD-1 occupancy with nivolumab was also confirmed in these circulating T cells (Fig. 4b).

The Olink proximity extension assay quantified 725 immuno-oncology-related proteins in serum samples collected before (day −4) and after (days +7 and +12) treatment with neoadjuvant ICI therapy. Most proteins (681 of 725; 94%) showed minimal changes in response to immunotherapy (log_2_ fold change after treatment relative to before treatment, between −1 and +1). A distinct subset (44; 6%) proteins showed an increase or decrease of greater than twofold at days +7 and +12, compared with pretreatment protein levels (Extended Data Table 4). These proteins were associated with T cell activation and cytokine and interleukin (IL) signaling; they included the drug targets LAG3 and PDCD1, several ILs (IL5, IL6, IL10, IL12), the C-X-C motif chemokine ligands CXCL9 and CXCL10, and IFNγ.

## Discussion

A single dose of neoadjuvant nivolumab, ipilimumab and relatlimab increased the diversity, abundance and activation of TILs in the posttreatment tumor compared with the baseline tumor in newly diagnosed *MGMT* promoter unmethylated GBM. In other settings, activation and expansion of TILs and peripheral T cells have been associated with an ICI response^21–23^. While we observed features indicative of an ICI response, we cannot conclude that they predict clinical benefit for this patient. The binding of nivolumab to TILs confirms that intravenously administered ICIs can access the parenchyma of a primary brain tumor, whether via direct penetration or T cell-bound trafficking. Seventeen months after neoadjuvant combined (triplet) ICI, a maximal safe resection, adjuvant immunotherapies and a standard course of adjuvant radiotherapy, the patient has no definitive evidence of recurrence, which exceeds the median prognosis for this GBM when treated with chemoradiotherapy^2,6^.

Our case highlights an opportunity to reexamine ICI for GBM with careful, data-driven and clinically informed optimization of neoadjuvant combination ICI in newly diagnosed GBM, without iatrogenic immunosuppression^24–27^. Our concern is that the negative ICI studies in GBM, using adjuvant single-agent ICI in pretreated patients with recurrent disease, may have missed a critical early ‘window of opportunity’^26,27^ where ICI is ideally positioned to achieve a potentially curative response. Single-agent anti-PD-1 was not effective in patients with recurrent GBM who received prior chemotherapy and corticosteroids (~40% of patients at baseline were on prednisone at doses <10 mg, probably reduced for ‘trial eligibility’ in CheckMate 143) (refs. ^12,28^). Likewise, concurrent anti-PD-1 and radiotherapy did not improve the outcomes of newly diagnosed patients with GBM with unmethylated *MGMT* promoter, where nearly 30% of patients were receiving corticosteroids at baseline^6^. This scenario mirrors that of patients with melanoma brain metastasis, where those requiring corticosteroids for symptom management have a poorer response to ICIs than those not requiring steroids^18,29^. Importantly, single-agent anti-PD-1 has demonstrated activity in isolated cases with GBM associated with germline mismatch repair deficiency^30,31^ and in newly diagnosed patients with *MGMT* methylated GBM treated in the neoadjuvant setting^12^.

While single-agent anti-PD-1 shows limited benefit in most patient cohorts with GBM, combination ICIs target multiple independent steps in the cancer–immunity cycle; when dosed optimally, they improved patient outcomes compared with anti-PD-1 alone in cancers such as melanoma^4^^,32^^,33^. A small feasibility study (*n* = 15, with nine patients unmethylated) reported a median OS of 19.3 months in newly diagnosed GBM treated with first-line adjuvant anti-PD-1 plus anti-CTLA-4, followed by standard radiotherapy^34^. One important barrier to investigating combination ICI is the induction of immune-related adverse events, which may affect quality of life, require immunosuppression that counteracts the activity of ICI or result in early cessation of ICI treatment. In our case study, combined (triplet) ICI was continued by personalizing the therapy to immune-related toxicity (delaying cycles and reducing combinations as necessary). Another perceived barrier to the use of combination ICI in brain tumors is the risk of cerebral edema; however, this was not observed in this case nor in patients with melanoma with active brain metastases who had no prior radiotherapy^18^. The perceived risk of using ICIs in GBM may be because of the concern of exacerbating radiotherapy-induced inflammation^35^, especially given the doses and volumes of radiotherapy typically used in this tumor.

There are limitations to this research, namely, that the clinical outcomes and biospecimens were derived from a single patient and technical replication was often not feasible because of the limited amount of sample. The generalizability of these findings may also be limited by the favorable early disease course, including a rapid interval between presentation and diagnosis, no tumor growth between the diagnostic and preoperative scans, good Eastern Cooperative Oncology Group Performance Status and limited tumor-associated edema (although there were some key poor prognostic factors, including an unmethylated *MGMT* promoter and incomplete resection because of the tumor’s proximity to eloquent brain regions). If neoadjuvant ICI were trialed in newly diagnosed patients with GBM, the regimen would need to be rationally adapted to individual tumor and clinical factors.

This case study suggests that neoadjuvant combination ICI can promote the infiltration, activation and expansion of tumor-specific T cells in newly diagnosed GBM. A regimen of upfront combination ICIs in newly diagnosed GBM is worthy of more thorough investigation before these agents can be excluded from the GBM treatment algorithm; a clinical trial, GIANT (trial registration no. NCT06816927), is planned.

## Methods

### Patient and ethics

This research included a single 56-year-old white patient of male sex and gender. It was conducted in accordance with the Declaration of Helsinki (version 2024) and CAse REports guidelines, and with written informed consent of the patient. All drug therapy used in this study was obtained from Bristol Myers Squibb and submitted to the Therapeutic Goods Administration, Australia (Special Access Scheme, Category A). There was no participant compensation.

Biospecimen samples were acquired with consent from the Sydney Brain Tumour Bank (no. 2019/ETH08929), the Melanoma Biospecimen Tissue Bank (no. HREC/11/RPAH/444) and the Macquarie University Cancer Biobank (no. HREC2793). Tumor sequencing was performed with consent from the Royal Melbourne Hospital Office for Research (no. HREC/61352/MH-2020 34).

### Next-generation sequencing and transcriptome analysis

WGS was performed on patient-matched normal and tumor DNA on the Illumina NovaSeq 6000 platform using the Illumina TruSeq Nano DNA library preparation kit according to the manufacturer’s instructions^38^. WGS analysis was performed using the Advanced Genomics Collaboration (TAGC) Clinical Genomics Analysis Platform to perform genome alignment (hg38) and variant calling using DRAGEN v.3.9 (refs. ^39,40^), copy number variation using PURPLE v.2.51 (ref. ^41^) and structural rearrangement detection via the DRAGEN SV and breakpointinspector v.1.5 packages and prioritized using the simple_sv_annotation^42^. All workflows are written in the Common Workflow Language and are freely available under the MIT license in a version-controlled repository (https://github.com/umccr/cwl-ica).

Total RNA samples were used as input for the NanoString IO 360 Panel and run on the nCounter Max/Flex Prep Station and Digital Analyzer^36^. Calculation of signature scores was performed using the singscore method from the raw read count table^36,37^.

### Flow cytometry analysis

Flow cytometry staining was performed on viable cryopreserved tumor or PBMC samples^10^. Samples were thawed and stained with fluorophore-conjugated antibodies against the following: CD45 (BUV737, clone HI30; 1/200 dilution, cat. no. 748719; research resouce ID (RRID): AB_2873123); CD45RA (BUV737, clone HI10; 1/100 dilution, cat. no. 564442; RRID: AB_2738810); CD45RO (BUV395, clone UCHL1; 1/20 dilution, cat. no. 564291; RRID: AB_2744410); CD3 (PE-CF594, clone UCHT1; 1/100 dilution, cat. no. 562280; RRID: AB_11153674); CD3 (BV786, clone UCHT1; 1/100 dilution, cat. no. 565491; RRID: AB_2739260); HLA-DR, HLA-DP, HLA-DQ (BUV395, clone Tu39; 1/200 dilution, cat. no. 740302; RRID: AB_2740041); CD8 (V500, clone SK1; 1/100 dilution, cat. no. 561617; RRID: AB_10896281); CD134 (OX40, PE-Cy7, clone Ber-ACT35; 1/20 dilution, cat. no. 563663; RRID: AB_2738358) (all were obtained from BD Biosciences); HLA-A, HLA-B, HLA-C (AF700, clone W6/32; 1/80 dilution, cat. no. 311438; RRID: AB_2566306); CD4 (AF700, clone A161A1; 1/40 dilution, cat. no. 357418; RRID: AB_2616933); HLA-DR (FITC, clone L243; 1/100 dilution, cat. no. 307604; RRID: AB_314682) (all from BioLegend); and CD223 (LAG3, PE, clone REA351; 1/11 dilution, cat. no. 130-105-452, Miltenyi Biotech; RRID: AB_2656407). Nonspecific staining was blocked with Fc block (clone Fc1, 1/200 dilution, cat. no. 564220, BD Biosciences; RRID: AB_2728082). For the detection of T cell-bound nivolumab, cells were incubated with human IgG4Fc (PE, clone HP6025; 1/100 dilution, cat. no. 9200-09, Southern Biotech; RRID: AB_2796693). For direct PD-1 detection, cells were stained with CD279 (PD-1, BV421, clone EH12.1; 1/50 dilution; cat. no. 562516, BD Biosciences; RRID: AB_11153482). Cell viability was determined by staining cells with LIVE/DEAD near-infrared fixable dye (cat. no. L34976, Thermo Fisher Scientific).

After cell surface staining, cells were fixed and permeabilized using the eBioscience Transcription Factor Buffer Kit (cat. no. 00-5523-00, Thermo Fisher Scientific) and stained with the following antibodies plus Fc block in permeabilization buffer: FOXP3 (PE-CF594, clone 236A/E7; 1/20 dilution, cat. no. 563955; RRID: AB_2738507); GFAP (AF488, clone 1B4; 1/20 dilution, cat. no. 560297; RRID: AB_1645350); GzmB (AF700, clone GB1; 1/100 dilution, cat. no. 560213; RRID: AB_1645453); SOX2 (PE, clone O30-678; 1/100 dilution, cat. no. 562195; RRID: AB_10895118) (all from BD Biosciences); CD152 (CTLA-4, APC, clone 14D3; 1/20 dilution, cat. no. 17-1529-42; RRID: AB_2688162); Ki-67 (APC, clone 20Raj1; 1/150 dilution, cat. no. 17-5699-42; RRID: AB_2573218) (both from Thermo Fisher Scientific). Samples were washed extensively and immediately acquired on a BD LSRFortessa X20 flow cytometer (BD Biosciences). Samples were analyzed with the FlowJo software v.10.8 (BD Biosciences).

### PhenoCycler–Fusion tissue imaging and analysis

Antibodies targeting tumor and non-tumor cells in the TME were conjugated to short DNA oligonucleotides and titrated according to the manufacturer’s instructions (Akoya Biosciences)^43^. Antigens shown in this study include the following: CD14 (AKYP0079, 1/200 dilution, Akoya Biosciences), CD11c (AKYP0051, 1/400 dilution, Akoya Biosciences), CD141 (AKP0124, 1/200 dilution, Akoya Biosciences), CD3 (AKYP0062, 1/200 dilution, Akoya Biosciences), CD8 (AKYP0028, 1/200 dilution, Akoya Biosciences), CD4 (AKYP0048, 1/200 dilution, Akoya Biosciences), GzmB (AKYP0086, 1/100 dilution, Akoya Biosciences), FOXP3 (AKYP0086, 1/100 dilution, Akoya Biosciences) and S100B (1/200 dilution, cat. no. 42397, Cell Signaling Technology). Initial T cell quantification was performed using the HALO AI v.3.6 image analysis platform with default artificial intelligence-based cell sequencing and manual gating of T cell phenotypes. Subsequent broad immunophenotyping was performed with cell segmentation in StarDist using the DAPI channel (cytoplasm segmentation was estimated using a morphological dilation of 5 µm); cell phenotyping for CD4^+^ and CD8^+^ T cells was performed using the machine learning classifier in QuPath v.0.4.4 (ref. ^44^). Cell neighborhood enrichment analysis was performed using a graph-based connectivity algorithm with the squidpy Python package^45^. Cell proximity in spatial neighborhoods was quantified with a permutation-based test (1,000 default) by comparing the spatial location of cell types and their relative distances.

### TCR pan-clonality assay

DNA target amplification (200 ng genomic DNA input), partial digestion, barcoding of amplicons and purification were performed according to the manufacturer’s protocol (Oncomine Human Immune Repertoire user guide for TCR pan-clonality assay, Thermo Fisher Scientific). The barcoding set consisted of the IonTorrent Dual Barcode Kit 1–96 (Thermo Fisher Scientific). Target amplification was for 31 cycles.

Purified libraries were quantified with quantitative PCR (qPCR) using an Ion Library TaqMan Quantitation Kit (Thermo Fisher Scientific) according to the Oncomine Human Immune Repertoire user guide. A 1/100 dilution of each sample library was analyzed in each case. qPCR was performed using a QuantStudio 7 Pro qPCR system (Thermo Fisher Scientific) in standard run mode (Oncomine Human Immune Repertoire user guide). Individual barcoded libraries were diluted to a final concentration of 50 pM in nuclease-free water using Eppendorf DNA LoBind microcentrifuge tubes (Sigma-Aldrich). For each sequencing chip, equal volumes (5 µl) of 12 diluted library samples were combined on ice. Subsequently, 25 µl of the pooled libraries were used for template preparation and chip loading.

The sequencing workflow consisted of template preparation and sequencing chip loading on an Ion Chef instrument (Thermo Fisher Scientific), sequencing on an Ion GeneStudio S5 plus system (Thermo Fisher Scientific) followed by data analysis. Planned sequencing runs were based on the Oncomine TCR Pan-Clonality Assay using the Torrent Suite software v.5.18.1. The kits used for each planned sequencing run were an Ion 550 Kit–Chef, Ion S5 Sequencing Kit and Ion 550 chip (Thermo Fisher Scientific). Sequencing data were automatically uploaded to the Ion reporter software v.5.18.4 for analysis using the Oncomine TCR Beta-SR and Gamma-SR–w1.4–DNA Single Sample workflow. Multi-sample analysis was based on join CDR3 nt.

Clonotypes were defined using V, J and CDR3 amino acid sequences from the TCRβ and TCRγ clone summaries from Thermo Fisher Scientific Ion Torrent targeted next-generation sequencing assay. Nonproductive clonotypes, based on the ‘functionality’ field, were excluded from the analysis. The productive clonotype frequency was calculated based on the total number of reads for a clonotype divided by the total number of productive reads in the sample. Clonotype diversity Shannon entropy (*H*) was calculated based on the clonotype abundances in each sample using *H* = −*Σpi* × ln(*pi*), where *pi* is the proportion of sequence *i* relative to the total N sequences^46^. Maximum Shannon diversity was calculated as ln(total clonotypes). The Shannon equitability or evenness was calculated as *H*/(max diversity).

### Distribution of clonotypes in each sample

Clonotypes were ranked according to their frequency, from largest to smallest, using min_rank from the dplyr R package (https://dplyr.tidyverse.org)^47^. The min_rank approach gives every tie the same (smallest) value. The cumulative frequency for the ranked clonotypes in each sample was calculated using the cumsum function from base R. Data analysis was performed in R v.4.3.0 (21 April 2023 release) using RStudio v.4.3.0 (https://cran.r-project.org/bin/windows/base/old/4.3.0/, ref. ^48^) with the following R packages: tidyverse v.2.0.0 (ref. ^49^); ggpubr (https://cran.r-project.org/web/packages/ggpubr/index.html); ggsci v.3.0.0 (https://github.com/nanxstats/ggsci); lemon v.0.4.9 (https://github.com/stefanedwards/lemon); and UpSetR v.1.4.0 (https://CRAN.R-project.org/package=UpSetR).

### Proximity extension assay

Plasma samples collected before (day −4) and after treatment (days +7 and +12) were analyzed using the proximity extension assay at the Olink Proteomics facility, Uppsala, Sweden. In total, 725 immuno-oncology-related protein biomarkers (Olink Explore 384 Inflammation, Olink Explore 384 Oncology (www.olink.com)) were measured and reported as normalized protein expression values, which is an arbitrary unit on a log_2_ scale, where a higher value corresponds to higher protein expression.

### CTC analysis

A total of 7.5 ml whole-blood samples were processed using the AccuCyte-CyteFinder platform (RareCyte)^50^. Nucleated blood cells were isolated using AccuCyte (RareCyte) and spread onto eight SuperFrost Plus slides (Thermo Fisher Scientific). Slides were immunofluorescently stained on the Autostainer Link 48 (Agilent Technologies) using the following: anti-GFAP (AF488; cat. no. 560297, Becton Dickinson), anti-EGFR (PE; cat. no. FAB9577P, R&D Systems), anti-CD45 (AF750; cat. no. NBP1-79127AF750, Novus Biologicals), anti-CD66b (AF750; cat. no. FAB42462, R&D Systems) antibodies and a nuclear DAPI dye (Thermo Fisher Scientific). Slides were then scanned on the CyteFinder HT (RareCyte) digital immunofluorescence microscope at ×10 magnification, with the following exposure times: 0.05 s (DAPI); 0.01 s (GFAP); 0.1 s (EGFR); and 0.1 s (CD45/CD66b). Image files were analyzed using an automated software and presented to the reviewer for CTC confirmation (CyteMapper, RareCyte). A CTC was defined as positive for DAPI and GFAP, and negative for CD45/CD66b (mean fluorescence intensity cutoff = 15).

### Reporting summary

Further information on research design is available in the Nature Portfolio Reporting Summary linked to this article.

## Online content

Any methods, additional references, Nature Portfolio reporting summaries, source data, extended data, supplementary information, acknowledgements, peer review information; details of author contributions and competing interests; and statements of data and code availability are available at 10.1038/s41591-025-03512-1.

## Supplementary information

Supplementary Information Supplementary Results 1–3.

Reporting Summary

## Source data

Source Data Figs. 2–5 and Extended Data Figs. 1–3 Statistical source data

## Figures and Tables

**Extended Data Table 1:**
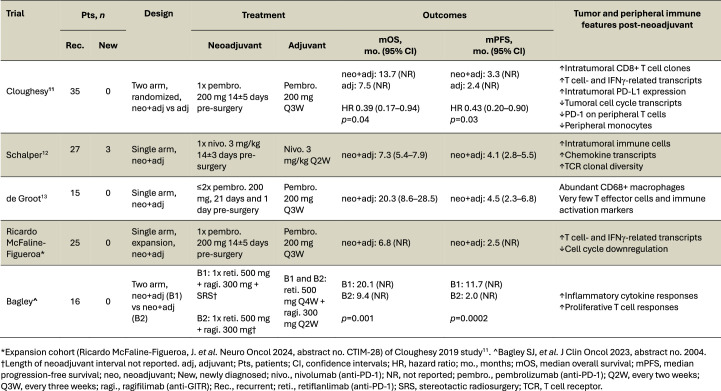
Neoadjuvant immune checkpoint inhibitor trials in glioblastoma

**Extended Data Table 2:**
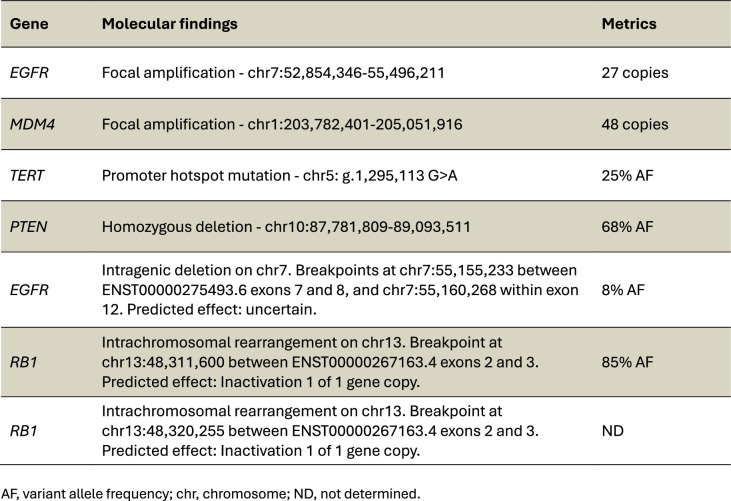
Whole-genome sequencing summary of the pretreatment specimen

**Extended Data Table 3:**
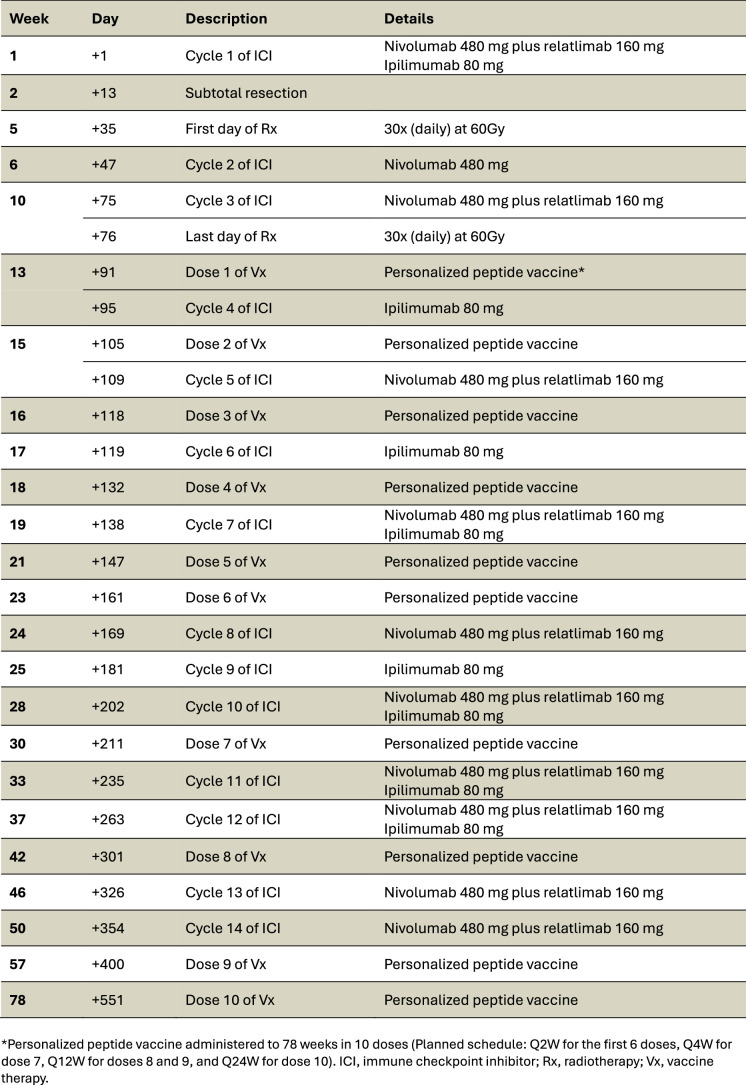
Chronological details of the treatment schedule

**Extended Data Table 4:**
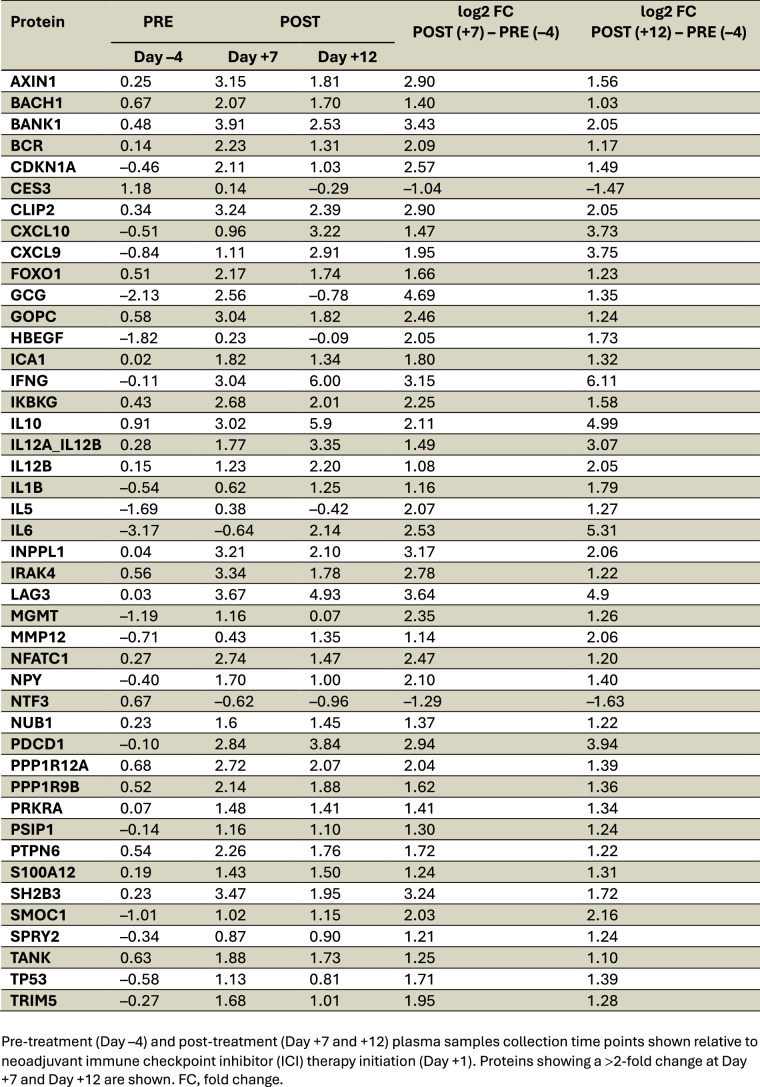
Normalized Olink proteomics plasma data expressed as normalized expression units on a log_2_ scale

## Data Availability

Requests for data access will be reviewed by the senior authors. Applicants can expect a response within 2 weeks of submission. Data will be provided under the following conditions: (1) the research must have received ethical approval from a recognized ethics review board; (2) the request must align with the scientific aims and goals of the dataset; (3) the requesting team must demonstrate the ability to handle the data securely and responsibly; (4) a formal data usage agreement must be signed, ensuring that the data will not be used for commercial purposes and will not be shared with unauthorized parties. Source data are provided with this paper.
