# Supplementary material for: Neoadjuvant triplet immune checkpoint blockade in newly diagnosed glioblastoma
Source: Nat Med. 2025 Feb 27;31(5):1557–66. doi: 10.1038/s41591-025-03512-1 (PMC12092302; doi:10.1038/s41591-025-03512-1)
Supplement: Supplementary file 1 — Supplementary Results 1–3. [file 41591_2025_3512_MOESM1_ESM.pdf]

nature medicine

Supplementary information https://doi.org/10.1038/s41591-025-03512-1

Neoadjuvant triplet immune checkpoint blockade in newly diagnosed glioblastoma

In the format provided by the authors and unedited

Supplementary Materials

Supplementary Results 1 | Patient presentation and initial treatment........1

Supplementary Results 2 | Pathology Report: initial biopsy specimen (Day –4)........2

Supplementary Results 3 | Pathology Report: safe maximal resection specimen (Day +13)........6

Supplementary-only References........8

Supplementary Results 1 | Patient presentation and initial treatment

The patient presented with a generalized seizure and was treated initially with intravenous (IV) diazepam. He underwent magnetic resonance imaging (MRI) brain, electroencephalogram (EEG), lumbar puncture, and standard blood analyses, which revealed a left temporal lobe pole mass (T2-weighted area measuring 48x23x26 mm with diffusion restriction and foci of contrast-enhancement measuring 7x3x5 mm), frontal intermittent rhythmic delta activity over the left temporal lobe and no pathogens. He was treated with levetiracetam (750 mg orally, twice daily) and IV aciclovir (750 mg three times daily for 14 days) for a possible differential diagnosis of viral encephalitis.

Supplementary Results 2 | Pathology Report: initial biopsy specimen (Day –4)

MACROSCOPIC DESCRIPTION

1. "LEFT TEMPORAL LESION"

A piece of tan tissue received, 15x9x6 mm

2. "LEFT TEMPORAL ENHANCING LESION"

Nine fragments of soft grey tissue, between 3 and 5 mm

MICROSCOPIC REPORT

Specimens 1 & 2 contain similar histological features and are described together. Both show a highly pleomorphic, diffusely infiltrative astrocytic glioma.

The tumor shows variable cellularity. In much of specimen 1, the tumor is moderately hypercellular, however, in some fragments in specimen 1 and in much of specimen 2 it shows marked hypercellularity. The tumor cells are pleomorphic, with a component of large atypical multinucleate giant cells. The tumor demonstrates variable morphology elsewhere, including areas where cells with gemistocytic morphology predominate, and others where there are intermediate sized atypical cells with astrocytic morphology. Rare foci of perivascular accumulation show some perivascular pseudorosette-like changes, although these changes are not widespread. Very focal microcalcification is seen in the form of psammoma-like bodies. Accompanying the tumor is a perivascular lymphocytic infiltrate noted in scattered intratumoural vessels.

The tumor involves both cortex and subcortical white matter. In the cortex, there is marked perineuronal satellitosis as well as subpial and perivascular accumulation. There is tumor encircling leptomeningeal vessels. This may represent focal leptomeningeal invasion, although this change is only seen at the surgical margin and may represent surgical artefact. Correlation with imaging and intraoperative findings is required.

There is no microvascular proliferation. There is no evidence of necrosis. Mitoses are present, with mitotic activity seen at up to 8–10 per 10 high-power fields (field diameter 0.58 mm).

Immunohistochemistry was performed and the results are as follows:

IDH1(R132H): negative
BRAF(V600E): negative
ATRX: retained nuclear staining (normal pattern)

NeuN: stains cortical pyramidal neurons

Synaptophysin: no significant tumor cell staining

GFAP: stains the majority of tumor cells

p53: moderate to strong nuclear staining in the majority of tumor cells, especially giant cells

EGFR: moderate cytoplasmic and variable membranous staining

H3K27M: negative

H3K27me3: retained nuclear staining (normal pattern)

Olig2: positive nuclear staining in many tumor cells

MAP2: strong background neuropil staining and variable staining of some tumor cells

CD163: highlights numerous parenchymal and perivascular microglia

CD68: stains some parenchymal and perivascular microglia/macrophages

CD3: positive staining of moderate numbers of perivascular lymphocytes and occasional parenchymal lymphocytes

CD20: stains roughly equal numbers of perivascular lymphocytes as CD3

CD4: stains the majority of CD3+ perivascular lymphocytes

CD8: stains a minority of perivascular CD3+ lymphocytes

CD34: scattered staining of occasional tumor cells, remote from areas of NeuN staining

Ki-67 labelling index (Blocks 1B & 2D): ~20–30%

Reticulin histochemistry: no increased parenchymal reticulin seen

Mismatch repair immunohistochemistry was also performed. The results are as follows:

MLH1: normal pattern of staining (retained nuclear staining)

PMS2: normal pattern of staining (retained nuclear staining)

MSH2: normal pattern of staining (retained nuclear staining)

MSH6: normal pattern of staining (retained nuclear staining)

*** Please note that the degree of staining meets the criteria for normal pattern of staining as defined in other sites, e.g. CAP protocol for Colon and Rectum Biomarker template 2018. Thresholds for staining have not yet been clearly defined in glioma; one recent paper describes a heterogenous pattern of MMR protein immunostaining in four of eight hypermutated gliomas.[1] Further investigation for the presence of microsatellite instability may be considered if clinically appropriate.

COMMENT

The tumor is a pleomorphic, mitotically active, diffusely infiltrative astrocytic glioma with negative IDH1(R132H) immunohistochemistry and retained nuclear ATRX stain. In a patient of this age, this staining pattern is indicative

of an *IDH*-wildtype diffuse astrocytic glioma. The presence of mitotic activity and the absence of necrosis and microvascular proliferation would be in keeping with a histological grade of 3.

Pyrosequencing for *TERT* promoter mutations and *IDH1* and *IDH2* variant mutations has been performed. No *IDH1* codon 132 or *IDH2* codon 172 mutation has been detected, however, there is a *TERT* promoter mutation (C228T). Therefore, the molecular phenotype of the tumor fulfils the CNS WHO 5 (2021) diagnostic criteria for a glioblastoma, *IDH*-wildtype, CNS WHO Grade 4.

The presence of variably prominent giant cells raises the possibility of a giant cell variant of an IDH-wildtype glioblastoma. There are some supportive features of this, namely perivascular lymphocytosis, focal perivascular pseudorosette-like structures, as well as strong p53 staining. However, the giant cells are not a “dominant” feature. Given the presence of giant cells, screening of mismatch repair enzyme deficiency was performed, however all four immunohistochemical stains showed a normal pattern of retained nuclear staining.

MGMT promoter methylation testing has been performed. The tumor is UNMETHYLATED at the *MGMT* promoter region.

Supplementary Results 3 | Pathology Report: safe maximal resection specimen (Day +13)

MACROSCOPIC DESCRIPTION

1. "LEFT TEMPORAL LESION FRESH"

A piece of tan tissue 35x30x8 mm with smooth cortex and leptomeninges seen. A few smaller fragments measuring in aggregate 6x4x2 mm

2. "LEFT AMYGDALA FRESH"

Two pieces of tan tissue, 8x4x2 mm and 6x4x2 mm as well as a small fragment 2 mm.

MICROSCOPIC REPORT

Specimens 1 & 2 contain similar histological features and are described together. The specimen was reviewed in conjunction with the previous resection.

The sections show cortex and subcortical white matter, in which the tumor demonstrates a spectrum of cellularity. The most cellular fragments appear morphologically similar to the previous resection, which comprises a minority of the specimen submitted. However there are very few giant/multinucleate tumor cells in this resection, and there are areas showing typical small cell morphology with associated microcalcifications, The remainder of the specimen shows less cellular but diffusely infiltrative tumor, with perineuronal satellitosis and prominent subpial aggregation noted.

Mitotic activity is difficult to find in the specimen. There is no necrosis or microvascular proliferation present.

Perivascular lymphocytic cuffing is present around many blood vessels. The cuffs are of similar or smaller size compared to the biopsy specimen. However immunohistochemistry for CD3 highlights significantly increased parenchymal T lymphocytes compared to the previous biopsy, which were difficult to identify on H&E-stained sections. In both perivascular and parenchymal locations the T lymphocytes are a mix of CD4 and CD8 subtypes, with CD4 slightly predominating over CD8 cells. CD20 highlights relatively similar numbers of B cells compared to the previous biopsy (less than CD3+ T cells), almost all in the perivascular cuffs. There are also small numbers of leptomeningeal lymphocytes present in this specimen, not seen in the previous biopsy.

CD163, CD68, and CD4 highlight a similar density of tumor-associated microglia/macrophages compared to the prior biopsy. The density is high in both specimens.

The previous operative site is represented in (1) – with some organizing tissue with macrophages.

Specimen 2 (left amygdala) is diffusely infiltrated by glioblastoma at low and moderate tumor cellularity, with morphologically similar lymphocytic infiltrate to Specimen 1.

EGFR immunohistochemistry was performed on blocks 2A and 2B (amygdala). There is strong and diffuse EGFR immunoreactivity seen in all tumor cells. This finding is consistent with the molecular identification of *EGFR* gene amplification.

Supplementary-only References

1 McCord, M. *et al.* The efficacy of DNA mismatch repair enzyme immunohistochemistry as a screening test for hypermutated gliomas. *Acta Neuropathol Commun* **8**, 15 (2020). https://doi.org:10.1186/s40478-020-0892-2
