# Supplementary material for: Neoadjuvant triplet immune checkpoint blockade in newly diagnosed glioblastoma
Source: Nat Med. 2025 Feb 27;31(5):1557–66. doi: 10.1038/s41591-025-03512-1 (PMC12092302; doi:10.1038/s41591-025-03512-1)
Supplement: Supplementary file 2 — Reporting Summary [file 41591_2025_3512_MOESM2_ESM.pdf]

nature portfolio

Corresponding author(s): Georgina V Long
Last updated by author(s): 09/01/2025

Reporting Summary

Nature Portfolio wishes to improve the reproducibility of the work that we publish. This form provides structure for consistency and transparency in reporting. For further information on Nature Portfolio policies, see our Editorial Policies and the Editorial Policy Checklist.

Statistics

For all statistical analyses, confirm that the following items are present in the figure legend, table legend, main text, or Methods section.

n/a	Confirmed	
☐	☒	The exact sample size (*n*) for each experimental group/condition, given as a discrete number and unit of measurement
☐	☒	A statement on whether measurements were taken from distinct samples or whether the same sample was measured repeatedly
☐	☒	The statistical test(s) used AND whether they are one- or two-sided<br>*Only common tests should be described solely by name; describe more complex techniques in the Methods section.*
☐	☒	A description of all covariates tested
☐	☒	A description of any assumptions or corrections, such as tests of normality and adjustment for multiple comparisons
☐	☒	A full description of the statistical parameters including central tendency (e.g. means) or other basic estimates (e.g. regression coefficient) AND variation (e.g. standard deviation) or associated estimates of uncertainty (e.g. confidence intervals)
☐	☒	For null hypothesis testing, the test statistic (e.g. *F*, *t*, *r*) with confidence intervals, effect sizes, degrees of freedom and *P* value noted<br>*Give P values as exact values whenever suitable.*
☒	☐	For Bayesian analysis, information on the choice of priors and Markov chain Monte Carlo settings
☐	☒	For hierarchical and complex designs, identification of the appropriate level for tests and full reporting of outcomes
☐	☒	Estimates of effect sizes (e.g. Cohen's *d*, Pearson's *r*), indicating how they were calculated

Our web collection on statistics for biologists contains articles on many of the points above.

Software and code

Policy information about availability of computer code

Data collection	Software used for data collection included: Oncomine TCR Pan-Clonality Assay using Torrent Suite software version 5.18.1. TCR data analysis was performed in R version 4.3.0 (2023-04-21) using RStudio version 4.3.0 (with the following R packages; tidyverse version 2.0.0 ggpubr, ggsci version 3.0.0, lemon version 0.4.9, and UpSetR version 1.4.0, dplyr, cumsum function from base R). CTC image files were analyzed by CyteMapper v3.11.2.40, RareCyte). PhenoCycler-Fusion T cell quantification was performed utilizing HALO AI 3.6 with default AI-based cell sequencing and manual gating of T cell phenotypes. Subsequent broad immunophenotyping was performed with cell segmentation within StarDist using the DAPI channel (cytoplasm segmentation was estimated by morphological dilation of 5 µm), and cell phenotyping for CD4+ and CD8+ T cells was performed using the machine learning classifier in QuPath v0.4.4. Cell neighborhood enrichment analysis was performed using a graph-based connectivity algorithm with the squidpy Python package. Flow cytometry samples were analyzed with FlowJo software v10.8 (BD Biosciences). WGS was performed using the Illumina NovaSeq 6000 platform. Clinical Genomics Analysis Platform was used to perform genome alignment (hg38) and variant calling using DRAGEN3.9, copy number variation using PURPLE, and structural rearrangement detection via DRAGEN SV and breakpointinspector packages. Total RNA samples were used as input for the NanoString IO360Panel and run on the nCounter MAX/FLEX Prep Station and Digital Analyzer. RNA signature scoring utilised the R (version 4.2.0) package singscore (1.16.0)
Data analysis	Software used for data analysis included: Illumina DRAGEN Bio-IT Platform v3.9 and DRAGEN Structural Variant (SV) Caller, and Python (hartwigmedical/pipeline5; packages: PURPLE, breakpointinspector) for NGS and transcriptome analysis; FlowJo v10.8 (BD Biosciences) for flow cytometry data; HALO AI v3.6, StarDist, QuPath v0.4.4, and Python (package: Squidpy) for PhenoCycler-Fusion analysis; R v4.3.0 (2023-04-21) with RStudio v4.3.0 (packages: tidyverse, ggpubr, ggsci, lemon, UpSetR) for Oncomine TCR Pan-Clonality assay analysis; and CyteMapper Analysis Software Suite v3.11.2.40 (RareCyte) for CTC analysis.

For manuscripts utilizing custom algorithms or software that are central to the research but not yet described in published literature, software must be made available to editors and reviewers. We strongly encourage code deposition in a community repository (e.g. GitHub). See the Nature Portfolio guidelines for submitting code & software for further information.

Data

Policy information about availability of data

All manuscripts must include a data availability statement. This statement should provide the following information, where applicable:

- Accession codes, unique identifiers, or web links for publicly available datasets
- A description of any restrictions on data availability
- For clinical datasets or third party data, please ensure that the statement adheres to our policy

Requests for data access will be reviewed by senior authors, and applicants can expect a response within two weeks of submission. Data will be provided under the following conditions: i) The research must have received ethical approval from a recognized ethics review board; ii) The request must align with the scientific aims and goals of the dataset; iii) The requesting team must demonstrate the ability to handle the data securely and responsibly; iv) A formal data usage agreement must be signed, ensuring the data will not be used for commercial purposes and will not be shared with unauthorized parties.

Research involving human participants, their data, or biological material

Policy information about studies with human participants or human data. See also policy information about sex, gender (identity/presentation), and sexual orientation and race, ethnicity and racism.

Reporting on sex and gender	The patient's biological sex and gender are both male, and both reported in the manuscript.
Reporting on race, ethnicity, or other socially relevant groupings	The patient's ethnicity/race is Caucasian, which is reported in the manuscript.
Population characteristics	One 56-year-old Caucasian Australian male.
Recruitment	The single patient was known to the primary author who conceived, designed, proposed, and administered the experimental neoadjuvant treatment regimen. The patient consented to participate in the n-of-one study, including the experimental treatment and collection and analysis of biospecimens.
Ethics oversight	This research was conducted in accordance with the Declaration of Helsinki. All drug therapy used in this study was obtained from Bristol-Myers Squibb, submitted to the Therapeutic Goods Administration, Australia (Special Access Scheme, Category A), and given with the consent of the patient. Biospecimen samples were acquired with consent from the Sydney Brain Tumour Bank (2019/ETH08929), the Melanoma Biospecimen Tissue Bank (HREC/11/RPAH/444), and the Macquarie University Cancer Biobank (HREC2793).

Note that full information on the approval of the study protocol must also be provided in the manuscript.

Field-specific reporting

Please select the one below that is the best fit for your research. If you are not sure, read the appropriate sections before making your selection.

☒ Life sciences ☐ Behavioural & social sciences ☐ Ecological, evolutionary & environmental sciences

For a reference copy of the document with all sections, see nature.com/documents/nr-reporting-summary-flat.pdf

Life sciences study design

All studies must disclose on these points even when the disclosure is negative.

Sample size	This was a unique case study involving a single patient (N=1) who was known to the authors prior to presentation and diagnosis of glioblastoma. Treatment began within two weeks of diagnosis. The value of longitudinal biospecimens in the setting of neoadjuvant immunotherapy was well-known to the primary author; hence, while this is a descriptive case study of the management of a single patient, biospecimen collection (including blood and tumor tissue) and analysis for immune changes were integrated with clinical management from the beginning. This allowed us to perform several hypothesis-generating analyses. Including more patients was not feasible, nor appropriate. A clinical trial to test this regimen in a larger cohort is currently being designed at the Peter MacCallum Cancer Centre, in Melbourne, VIC, Australia.
Data exclusions	There were no data exclusions.
Replication	There were no technical replicates in the study, given the limited and irreplaceable biospecimens that required fractionation with different processing methods (e.g., tumor dissociates, fresh frozen tissue, FF/PE, etc).
Randomization	As this was a single patient study (N=1), no randomization was performed.
Blinding	The study was open-label and non-comparative. There was no blinding of any persons involved in the study, including the patient.

Reporting for specific materials, systems and methods

We require information from authors about some types of materials, experimental systems and methods used in many studies. Here, indicate whether each material, system or method listed is relevant to your study. If you are not sure if a list item applies to your research, read the appropriate section before selecting a response.

Materials & experimental systems

n/a	Involved in the study
☐	☒ Antibodies
☒	☐ Eukaryotic cell lines
☒	☐ Palaeontology and archaeology
☒	☐ Animals and other organisms
☒	☐ Clinical data
☒	☐ Dual use research of concern
☒	☐ Plants

Methods

n/a	Involved in the study
☒	☐ ChIP-seq
☐	☒ Flow cytometry
☐	☒ MRI-based neuroimaging

Antibodies

Antibodies used

Flow cytometry:
1. CD45 BUV737; 1:200; clone HI30, Cat. 748719; BD Biosciences; RRID AB_2873123
2. CD45RA BUV737; 1:100; clone HI100, Cat. 564442; BD Biosciences; RRID AB_2738810
3. CD45RO BUV395; 1:20; clone UCHL1, Cat. 564291; BD Biosciences; RRID AB_2744410
4. CD3 PE-CF594; 1:100; clone UCHT1, Cat. 562280; BD Biosciences; RRID AB_11153674
5. CD3 BV786; 1:100; clone UCHT1, Cat. 565491; BD Biosciences; RRID AB_2739260
6. HLA-DR, DP, DQ BUV395; 1:200; clone Tu39, Cat. 740302; BD Biosciences; RRID AB_274004
7. CD8 V500; 1:100; clone SK1, Cat. 561617; BD Biosciences; RRID AB_10896281
8. CD134 PE-Cy7; 1:20; clone Ber-ACT35, Cat. 563663; BD Biosciences; RRID AB_2738358
9. HLA-A, B, C AF700; 1:80; clone W6/32, Cat. 311438; BioLegend; RRID AB_2566306
10. CD4 AF700; 1:40; clone A161A1, Cat. 357418; BioLegend; RRID AB_2616933
11. HLA-DR FITC; 1:100; clone L243, Cat. 307604; BioLegend; RRID AB_314682
12. CD223 PE; 1:11; clone REA351, Cat. 130-105-452, Miltenyi Biotech; RRID AB_2656407
13. Fc block 1:200; clone Fc1, Cat. 564220; BD Biosciences; RRID AB_2728082
14. Human IgG4Fc PE; 1:100; clone HP6025, Cat. 9200-09; Southern Biotech; RRID AB_2796693
15. CD279 BV421; 1:50; clone EH12.1; Cat. 562516; BD Biosciences; RRID AB_11153482
16. FOXP3 PE-CF594; 1:20; clone 236A/E7; Cat. 563955; BD Biosciences; RRID AB_2738507
17. GFAP AF488; 1:20; clone 1B4; Cat. 560297; BD Biosciences; RRID AB_1645350
18. Granzyme B AF700; 1:100; clone GB11; Cat. 560213; BD Biosciences; RRID AB_1645453
19. SOX2 PE; 1:100; clone O30-678; Cat. 562195; BD Biosciences; RRID AB_10895118
20. CD152 APC; 1:20; clone 14D3; Cat. 17-1529-42; ThermoFisher Scientific; RRID AB_2688162
21. KI-67 APC; 1:150; clone 20Raj1; Cat. 17-5699-42; ThermoFisher Scientific; RRID AB_2573218

PhenoCycler-Fusion:
22. CD14 BX037; 1:200; clone AKYP0079, Cat. 4450047; Akoya Biosciences; RRID AB_3083457
23. CD11c BX024; 1:400; clone AKYP0051, Cat. 4550114; Akoya Biosciences; RRID AB_3083459
24. CD141 BX087; 1:200; clone AKYP0124, Cat. 4250097; Akoya Biosciences; RRID AB_3082975
25. CD3e BX045; 1:200; clone AKYP0062, Cat. 4550119; Akoya Biosciences; RRID AB_2936080
26. CD8 BX026; 1:200; clone AKYP0028 C8/114B, Cat. 4250012; Akoya Biosciences; RRID AB_2915960
27. CD4 BX003; 1:200; clone AKYP0048 EPR6855, Cat. 4550112; Akoya Biosciences; RRID AB_3094499
28. S100B BX049 (self-conjugated); 1:200; clone E7C3A, Cat. 42397SF; Cell Signalling Technology

Circulating tumor cells:
29. GFAP AF488; 1:50; clone 1B4, Cat 560297; BD Pharmingen; RRID AB_1645350
30. GFR PE; 1:100; clone Hu1; Cat. FAB9577P; R&D Systems; RRID AB_2942015
31. CD45 AF750; 1:100; clone HI30; Cat. NBP1-79127AF750; Novus Biologicals; RRID AB_309771
32. CEACAM-8/CD66b AF750; 1:100; clone 913542; Cat. FAB4246S; R&D Systems; RRID AB_3097716

We used healthy donors peripheral blood cells and glioblastoma tumor dissociates and cell lines to confirm the staining specificity and determine dilutions.

Validation

Antibodies 1 through 21 were validated by the relevant manufacturer as human-reactive (CQ testing or routinely tested) for application in flow cytometry. Additional manufacturer information: 9. Verified reactivity in human, cynomolgus, rhesus. 11. Verified reactivity in human, cynomolgus, rhesus. 17. Application "bioimaging". 20. Reactivity in human and rhesus. 21. Reactivity in human and dog. Antibodies 22 through 27 were developed by the manufacturer specifically for use in PhenoCycler-Fusion (PhenoCode Discovery) with human-reactivity. Antibody 28 is a carrier-free primary antibody validated as reactive in humans, mice, and rats by the manufacturer. It is designed for conjugation with fluorophores, metals, lanthanides, and oligonucleotides. The antibody was conjugated in-house to BX049 AlexaFluor 750 for PhenoCycler. Antibodies 29 through 32 were validated as human-reactive for bioimaging (29, 31) and/or flow cytometry (30, 32).

Flow Cytometry

Plots

Confirm that:

☒ The axis labels state the marker and fluorochrome used (e.g. CD4-FITC).

☒ The axis scales are clearly visible. Include numbers along axes only for bottom left plot of group (a 'group' is an analysis of identical markers).

☒ All plots are contour plots with outliers or pseudocolor plots.

☒ A numerical value for number of cells or percentage (with statistics) is provided.

Methodology

Sample preparation	Viable cryopreserved tumor or PBMC samples were thawed, washed and immediately stained with fluorophore-conjugated antibodies. Non-specific staining was blocked with Fc block. For the detection of T-cell bound nivolumab, cells were incubated with human IgG4Fc-PE. For indirect PD1 detection, samples were incubated with pembrolizumab (20μg/ml, Merck) prior to staining with fluorophore-conjugated antibodies, and cell-bound drugs (both IgG4) were detected with IgG4Fc-PE. For direct PD1 detection, cells were stained with CD279-BV421. Cell viability was determined by staining cells with LiveDead near-infrared (NIR) fixable dye. After cell surface staining, cells were fixed and permeabilized using the eBioscience transcription factor buffer kit, and stained with antibodies against intracellular markers plus Fc block in permeabilization buffer. Samples were washed extensively and immediately acquired on the flow cytometer.
Instrument	5-laser BD LSRFortessa X20 flow cytometer (BD Biosciences)
Software	FlowJo v10.8 (BD Biosciences)
Cell population abundance	No cell sorting was performed in this study
Gating strategy	The gating strategy involved: an FSC vs SSC gate to exclude debris, a time gate to exclude electronic noise, a viability gate (LiveDead NIR-negative) to exclude dead cells, a singlet gate (FSC height vs FSC area) to exclude doublets, and tumor and immune fraction gates to define the cells of interest. Tumor cells (SOX2+) were further gated for the GFAP+ fraction and analyzed for MHC class I (HLA-A, B, C) and MHC class II (HLA-DR, DP, DQ) expression. Tumor-infiltrating CD45+ (immune) fraction was analyzed for T cell content (CD3+), T cell subsets (CD8+ T, CD4+FOXP3- Tem and CD4+FOXP3+ Tregs) and immune checkpoints (CD39, LAG3, TIGIT, TIM3, and CTLA4).<br><br>GBM cells: CD45-_SSC-A int to high_SOX2+_GFAP+.<br>TILs: CD45+_SCC-A low_CD3+.<br>CD8 effector/memory cells: CD45+_CD3+ SSC-A low_CD8+_CD45RA- CD45RO+.<br>CD4 effector/memory cells: CD45+_CD3+ SSC-A low_CD4+FOXP3-_CD45RA- CD45RO+.<br>Tregs: CD45+_CD3+ SSC-A low_CD4+FOXP3+_ CD45RA-CD45RO+

☒ Tick this box to confirm that a figure exemplifying the gating strategy is provided in the Supplementary Information.

Magnetic resonance imaging

Experimental design

Design type	MRI Brain. Note that MRI was used as a routine diagnostic and monitoring tool, and was not experimental.
Design specifications	Brain Tumour Protocol
Behavioral performance measures	Nil

Acquisition

Imaging type(s)	MRI brain, with Siemens Healthineers MAGNETOM Vida
Field strength	3 Tesla
Sequence & imaging parameters	3D T2 FLAIR, 3D T1 MPRAGE, 3D MPRAGE C+, Ax T2, DWI, SWI, DSC Perfusion
Area of acquisition	Head - brain

Diffusion MRI ☒ Used ☐ Not used

Parameters: Nil

Preprocessing

Preprocessing software	Siemens
Normalization	Siemens Inline
Normalization template	Siemens Inline
Noise and artifact removal	Siemens Inline
Volume censoring	Siemens Inline

Statistical modeling & inference

Model type and settings	Dynamic Susceptibility Contrast
Effect(s) tested	Perfusion - CBV

Specify type of analysis: ☒ Whole brain ☐ ROI-based ☐ Both

Statistic type for inference (See Eklund et al. 2016)	Perfusion Metrics
Correction	Nil

Models & analysis

n/a	Involved in the study
☒	☐ Functional and/or effective connectivity
☒	☐ Graph analysis
☒	☐ Multivariate modeling or predictive analysis
